# High throughput cultivation-based screening on porous aluminum oxide chips allows targeted isolation of antibiotic resistant human gut bacteria

**DOI:** 10.1371/journal.pone.0210970

**Published:** 2019-01-17

**Authors:** Dennis Versluis, Teresita de J. Bello González, Erwin G. Zoetendal, Mark W. J. van Passel, Hauke Smidt

**Affiliations:** 1 Laboratory of Microbiology, Wageningen University & Research, Wageningen, the Netherlands; 2 Center for Infectious Disease Control, National Institute for Public Health and the Environment, Bilthoven, the Netherlands; Colorado State University, UNITED STATES

## Abstract

The emergence of bacterial pathogens that are resistant to clinical antibiotics poses an increasing risk to human health. An important reservoir from which bacterial pathogens can acquire resistance is the human gut microbiota. However, thus far, a substantial fraction of the gut microbiota remains uncultivated and has been little-studied with respect to its resistance reservoir-function. Here, we aimed to isolate yet uncultivated resistant gut bacteria by a targeted approach. Therefore, faecal samples from 20 intensive care patients who had received the prophylactic antibiotic treatment selective digestive decontamination (SDD), i.e. tobramycin, polymyxin E, amphotericin B and cefotaxime, were inoculated anaerobically on porous aluminium oxide chips placed on top of poor and rich agar media, including media supplemented with the SDD antibiotics. Biomass growing on the chips was analysed by 16S rRNA gene amplicon sequencing, showing large inter-individual differences in bacterial cultivability, and enrichment of a range of taxonomically diverse operational taxonomic units (OTUs). Furthermore, growth of *Ruminococcaceae* (2 OTUs), *Enterobacteriaceae* (6 OTUs) and *Lachnospiraceae* (4 OTUs) was significantly inhibited by the SDD antibiotics. Strains belonging to 16 OTUs were candidates for cultivation to pure culture as they shared ≤95% sequence identity with the closest type strain and had a relative abundance of ≥2%. Six of these OTUs were detected on media containing SDD antibiotics, and as such were prime candidates to be studied regarding antibiotic resistance. One of these six OTUs was obtained in pure culture using targeted isolation. This novel strain was resistant to the antibiotics metrodinazole and imipenem. It was initially classified as member of the *Ruminococcaceae*, though later it was found to share 99% nucleotide identity with the recently published *Sellimonas intestinalis* BR72^T^. In conclusion, we show that high-throughput cultivation-based screening of microbial communities can guide targeted isolation of bacteria that serve as reservoirs of antibiotic resistance.

## Introduction

The emergence of bacterial pathogens that are resistant to clinical antibiotics is an increasing threat to public health. A common route through which pathogens can acquire resistance is by genetic exchange with human-associated bacteria, and especially the gut microbiota [1–3]. Indeed, it has been shown that the commensal gut microbiota harbours diverse resistance genes [4, 5], that these genes are regularly expressed [6] and that such genes can be acquired by (opportunistic) pathogens [7]. Horizontal gene transfer (HGT) is considered the main mechanism by which resistance genes are disseminated, and it has been shown that HGT events occur exceedingly more often in the gut microbiota than in other environments with complex bacterial communities [8].

Though novel resistance determinants are typically described once bacteria are obtained in pure culture, resistance genes carried by yet uncultivated gut bacteria appear to be largely uncovered. This perspective is reinforced by the observation that functional metagenomics studies of human gut microbiota consistently yield novel resistance genes [9, 10]. This “black box” of little-studied uncultivated bacteria has been estimated to constitute 40–70% of gut microbes [11, 12]. Even though application of culture-independent methods (e.g. metagenomics [13, 14]) has provided us with useful insights into uncultivated bacteria and their resistance genes, their cultivation will be essential to comprehensively study the antibiotic resistance phenotype, and the potential roles and mechanisms of these bacteria in antibiotic resistance dissemination.

Nowadays, to isolate members of yet uncultivated taxa, innovative culturing techniques that apply high-throughput screening and/or better simulate the natural environment of these bacteria are increasingly being used. Recent methodical advances include cultivation inside chambers placed in the native environment [15–17], the use of custom-designed media [18, 19], application of multi-well micro culture chips [20], high-throughput identification of isolates [21], and microfluidic cultivation [22]. Furthermore, a recent study by Rettedal and co-authors combined high-throughput sequencing with selective cultivation conditions, allowing cultivation of previously uncultured species from the human gut by a targeted approach [23]. To this end, the authors used, among other criteria, the “most wanted” list of microbial taxa that has recently been introduced in order to guide efforts towards the cultivation of human gut bacteria [24]. In short, the most wanted list contains human-associated bacterial taxa of which the genome has not yet been sequenced, not considering whether members of these taxa from other environments might already have been sequenced. High priority most wanted taxa were defined as those of which the 16S ribosomal RNA (rRNA) centroid read shared less than 90% identity with either the GOLD-Human or Human Microbiome Project (HMP) strains, and which were detected in at least 20% of samples from any body habitat analysed. Medium priority taxa are those that share between 90% and 98% identity with the same habitat prevalence threshold. Antibiotic resistance has not played a role in the determination of the most-wanted list, though resistance features in these species may be relevant to the dissemination of resistance in the human gut.

In most Dutch hospitals, patients who are admitted to the intensive care unit (ICU) receive prophylactic antibiotic therapies, of which selective decontamination of the digestive tract (SDD) is currently the most common treatment. SDD combines the application of tobramycin, polymyxin E and amphotericin B in the oropharynx and gastrointestinal tract with a short systemic administration of a third-generation cephalosporin. The therapy aims to eradicate potential pathogens such as *Staphylococcus aureus*, *Pseudomonas aeruginosa*, *Enterobacteriaceae* and fungi while maintaining the anaerobic members of the microbiota [25]. SDD therapy has been shown to decrease infections and mortality of ICU patients [26, 27]. Although a meta-analysis showed that SDD therapy resulted in a decrease in resistance carriage with respect to cultivable bacteria [28], a recent case study [14] and a more extensive follow-up with 13 ICU patients [29] indicated that prophylactic therapy may in fact increase resistance gene carriage among mostly uncultivated anaerobic gut residents. It was speculated that the expanded resistome, i.e. the collection of all resistance genes in a bacterial community [30], might thereby increase the risk of potential pathogens becoming resistant in the future. Indeed, the risk that pathogens develop antibiotic resistance is a major concern that has prohibited wide implementation of prophylactic therapies [28]. In view of the above, it is clear that the role of uncultivated anaerobic bacteria in the emergence of resistance pathogens merits deeper investigation.

In this study, we aimed to isolate with a targeted approach potential antibiotic resistance reservoir species from the human gut microbiota under anaerobic conditions. We applied porous aluminium oxide (PAO) chips as substrate for bacterial growth to reduce potential toxicity of agar and to facilitate efficient parallel processing of a large number of samples [20].

## Materials and methods

### Sample collection

Faecal samples were collected from 20 patients on average five days after admission to the ICU at Utrecht Medical Center, Utrecht, Netherlands (S1 Table). During this period, the patients received SDD therapy [14]. The patients were not exposed to the SDD antibiotics in the 12 months prior to admission. The SDD protocol was reviewed and approved by the institutional review board of the University Medical Center Utrecht under number 10/0225 while written consent for the collection of faecal samples was obtained after hospitalization [14]. Faecal samples were collected upon defecation and stored at 4°C for 30 min to 4 h, after which an aliquot of the sample (approximately 0.5 g) was suspended in 5 ml of anoxic phosphate-buffered saline (PBS) (pH 7.0). Subsequently, 1 ml of the suspension was transferred into an anoxic bottle containing 4 ml of PBS, 25% (v/v) glycerol, 0.5 g resazurin and 0.5 g cysteine. To preserve anoxic conditions, a few drops of titanium citrate (100 mM) were added to the bottle before storage at -80°C.

### Bacterial cultivation

A high throughput cultivation technique using PAO chips (brand name Anopore, General Electric, Germany) was applied. Faecal bacteria were cultured on ethanol-sterilized PAO chips on top of two different media: (i) Gifu anaerobic agar medium (GAM) (Hyserve, Uffing, Germany), and (ii) bicarbonate-buffered anoxic medium (referred to in the text as CP medium) [31] supplemented with 1.5% (w/v) agar and 1% (v/v) faecal supernatant. The faecal supernatant was prepared from a pool of faecal samples obtained from three healthy volunteers who had not received antibiotics for at least six months. In brief, equal amounts of faecal sample from the three volunteers were added to anoxic PBS (pH 7.0) to a final concentration of 25% (w/v). Subsequently, the mixture was centrifuged at 14,000 rpm for 30 min, after which the supernatant was transferred to an anoxic bottle (N_2_/CO_2_−80:20, v/v) and autoclaved. The GAM and CP agar media were prepared both with and without the addition of the SDD cocktail of antibiotics (25 μg/ml tobramycin, 5 μg/ml polymyxin E and 10 μg/ml cefotaxime; the antifungal drug amphotericin B was not included in this study).

Five μl undiluted, 10-fold diluted, and 100-fold diluted cryopreserved faecal suspension from each of the 20 patients was inoculated in duplicate (biological duplicates) on PAO chips that were placed on top of GAM and CP agar media with and without the SDD antibiotic cocktail. PAO chips on top of GAM agar and CP agar were harvested two and three days after inoculation under anoxic conditions at 37°C, respectively. To prevent contamination, we used good microbiological practice, and worked in an anaerobic tent. However, microbial contamination via the tent atmosphere can never be completely eliminated. Upon harvesting, PAO chips with bacterial growth were placed in an Eppendorf tube containing 1 ml of anoxic PBS (pH 7.0). The tube was vortexed for 30 s to dissociate the cells from the PAO chip. Subsequently, the suspension was split into two fractions; one fraction was used for DNA extraction whereas the other fraction was added to an anoxic bottle containing glycerol (final concentration: 25–30%) in PBS, and stored at -80°C.

### 16S rRNA gene amplicon sequencing

#### DNA extraction

16S rRNA gene amplicon sequencing was used to investigate the bacterial composition of the cryo-preserved faecal samples and of the associated bacterial growth communities (suspended in PBS, see above). Microbial diversity on PAO chips inoculated with undiluted and 100-fold diluted faecal sample was analysed for all 20 patient whereas microbial diversity on PAO chips inoculated with 10-fold diluted faecal sample was analysed only for patients 131, 148 and 210. First, the cells in these samples were lysed and (cellular) debris was removed with an adapted bead beating protocol [32].

In case of cryo-preserved faecal material, 500 μl of sample was added to a screw-cap tube that already contained 0.5 g of 0.1 mm zirconium beads (Biospec Products, Bartlesville, United States) and three 5 mm glass beads (Biospec Products). Subsequently, 300 μl Stool Transport, and Recovery (STAR) buffer (Roche, Basel, Switzerland) was added, after which the contents of the tube were homogenized in the Precellys 24 (Bertin Technologies, Montigny-le-Bretonneux, France) at 5.5 ms (3 rounds of 1 min). The sample was then incubated at 95°C at 100 rpm for 15 min. Particles were spun down at 4°C at >10,000 g for 5 min, and subsequently the supernatant was transferred to a fresh tube for DNA extraction. The DNA yield was improved by another two iterations of bead beating that started with re-suspending the pellet in 300 μl STAR buffer.

In case of bacteria suspended in PBS (i.e. the growth communities), 150 μl of sample was processed by identical methods except at a smaller scale. Therefore, 0.25 g of 0.1 mm zirconium beads and three 2.5 mm glass beads were added to the screw-cap tube, and STAR buffer was used in portions of 150 μl.

Following the bead beating protocol, 250 μl aliquots of the combined supernatants were transferred into a Maxwell 16 Tissue LEV total RNA Purification Kit cartridge customized for DNA extraction (XAS 1220) (Promega, Madison, United States) [33]. The final extraction steps were carried out by the Maxwell 16 Instrument according to the manufacturer’s instructions. The quantity and quantity of the DNA was assessed using a NanoDrop ND-1000 spectrophotometer (NanoDrop Technologies, Wilmington, United States)

#### PCR and library preparation

16S rRNA gene amplification, which also attached the barcodes, was done with a 2-step PCR protocol [34] of which the primers are listed in S2 Table. The forward 27F-DegS primer and the equimolar mix of reverse primers 338-R-I and 338R-II were previously described in the context of the study of human faecal bacteria [35–37]. The product from the second PCR step was analysed on a 1% agarose gel and purified using the CleanPCR Kit (GC Biotech, Alphen aan den Rijn, Netherlands) according to manufacturers’ instructions. In each PCR run, a no template control was included to monitor environmental contamination through the presence or absence of a PCR product. The DNA concentration was measured by Qubit 2.0 (Thermo Fisher Scientific). Subsequently the sample was included in a pool that in total contained 48 equimolarly mixed samples. The pool of samples, which constituted a library, was sent for Illumina paired end MiSeq sequencing (2 x 300 bp) at GATC Biotech (Constance, Germany). In total, eight libraries were sent for MiSeq sequencing. Amplification product pertaining to cryo-preserved faecal samples was sequenced twice (i.e. technical replicates).

### Processing of 16S rRNA gene amplicon data

The 16S rRNA gene amplicon data were analysed using the NG-tax pipeline [38]. Default settings were used unless mentioned otherwise. The forward and reverse primers of each sample contained an identical 8-nt barcode. Therefore, barcodes needed to match in order for reads to be retained. OTUs were picked using the script OTU_picking_pair_end_read.sh with the minimal threshold for detectable OTUs set at 0.1% abundance, the error clustering percentage set at 0.985, and both the length of the forward and the reverse read set at 100. OTUs were filtered for plausible chimaeras according to the principle outlined by the developers of NG-tax [38]. The OTUs were annotated using SILVA release 111 [39] as a reference. The quality of the sequencing was controlled by including a mock community sample in each library. The mock community, which consisted of a fixed ratio of 16S rRNA amplicon DNA of different bacterial species (S3 Table), was developed in-house [38]. The sample preparation for the mock community started at the first PCR step, after which it was treated identical to the other samples. The output OTU table and centroid OTU sequences were used as input for detection of most wanted [24] and novel species. For statistical analyses, we rarefied the OTU table to 2,500 reads per sample with the QIIME [40] script single_rarefaction.py. Samples with less than 2,500 reads were excluded.

### Statistical analyses

Bray-Curtis dissimilarity indices between bacterial communities in inocula (i.e. faecal material of ICU patients) and their respective growth communities were calculated based on OTU-level data using QIIME. Shannon diversity, richness and phylogenetic diversity whole tree metrics of bacterial communities were also calculated using QIIME. The two-tailed t-test without assuming equal variance was used to investigate if Shannon, richness and whole-tree phylogenetic diversity values of bacterial communities differed significantly when grouped according to experimental variables (e.g. growth medium or supplementation of the medium with antibiotics). The t-test used averaged values for biological and technical replicates. The QIIME script *compare_taxa_summaries*.*py* was used to calculate Pearson correlation coefficients of OTU-level taxa between mock communities and their theoretical composition. Canonical Correspondence Analysis (CCA) as implemented in Canoco 5 [41] was used to investigate, which variables could best explain the variation in bacterial composition between bacterial communities. Linear mixed-effect models were fitted by the R package “lmerTest” (https://CRAN.R-project.org/package=lmerTest) [42] in order to analyse how media type and addition of antibiotics affected bacterial composition. As input an adapted OTU table was used in which values were log1p transformed to meet normality assumptions. Furthermore, OTUs were removed from the table if they were detected in <5 samples or by <50 reads across all samples. Parameter-specific *p*-values were obtained by using the Satterthwaite approximation. P-values were corrected for multiple testing by the function p.adjust in the package “stats”, using methods “Bonferroni” and “BH”. Bray-Curtis dissimilarity hierarchical clustering was performed using R package ‘Vegan’ based on OTU-level relative abundance data of bacterial communities.

### Detection of most wanted and novel species

In order to investigate the presence of most wanted taxa [24] and novel species, the representative reads of the OTUs were compared by Blastn [43] to the V1-V3 sequence data of the most wanted OTUs, and to the 16S rRNA genes in the SILVA database of cultured type strains (downloaded on May 19, 2015) [39], respectively. Custom Perl scripts were used to parse the BLAST results for the best hits (bitscore sorted) (S1 and S2 Texts). Furthermore, the script tabulated the relative abundance of the OTUs and their distribution across all samples.

### Targeted cultivation

Based on analysis of the 16S rRNA gene sequence data of the bacterial growth communities, OTUs were selected for targeted isolation. Therefore, the original faecal inoculum and the enriched growth fractions that contained the target OTU were re-plated under identical conditions, i.e. on PAO chips placed on the same media. A dilution series was inoculated to yield single colonies. Per PAO chip, three colonies per unique colony morphology were transferred to a fresh PAO chip. Subsequently, the 16S rRNA gene was amplified using the 27F and 1492R primers [44], and the PCR products were Sanger sequenced at GATC Biotech (Cologne, Germany) using the 907R primer [45]. The 16S rRNA gene sequences were compared by BLASTn to those in the 16S ribosomal RNA sequences (Bacteria and Archaea) database for species identification. Only for target species the near full-length 16S rRNA gene was then Sanger sequenced using the 27F and 1492R primers.

### Antibiotic susceptibility of target isolate

Antibiotic susceptibility of a pure culture isolate (i.e. a distinct colony was re-streaked three times) obtained by targeted isolation was tested using ATB ANA EU (08) (Biomerieux, Nürtingen, Germany) according to the manufacturer’s instructions, where bacterial growth in the presence of antibiotics was tested in ATB S Medium (BioMérieux) following the CLSI M11-A8 and M100-S24 breakpoints [46–48]. In brief, fresh colonies (3–5 colonies) from GAM agar media (after 48 h of growth) were picked with a plastic loop and introduced into the growth medium until a turbidity pertaining to the McFarland no. 3 standard (9 x 10^8^ CFU/ml) was achieved. From this suspension, 200 μl was added to 7 ml of ATB S medium, and 135 μl of this suspension was subsequently added to each well of the ATB ANA strip that contained one of the following (combinations of) antibiotics: penicillin G, amoxicillin, amoxicillin and clavulanic acid, ticarcillin, ticarcillin and clavulanic acid, piperacillin, piperacillin and clavulanic acid, imipenem, cefoxitin, cefotaxime, clindamycin, moxifloxacin, vancomycin, rifampicin, chloramphenicol, or metrodinazole. Finally, the panel was incubated in an anaerobic chamber with an atmosphere of N_2_/CO_2_ 80: 20 for 24 h. The interpretation of the results was performed according to the manufacturer’s instructions. Furthermore, a microdilution test was performed on 96 well plates using GAM broth instead of Wilkins-Chalgren medium in order to test the minimal inhibitory concentration (MICs) of tetracycline, ceftriaxone, ampicillin, imipenem and metronidazole in a dilution series ranging from 0.25 to 256 μg/ml dissolved antibiotic. *Clostridium innocuum* DSMZ 1286^T^ was included as a negative control for the microdilution test.

## Results

### Bacterial growth on PAO chips

**Fig 1:**
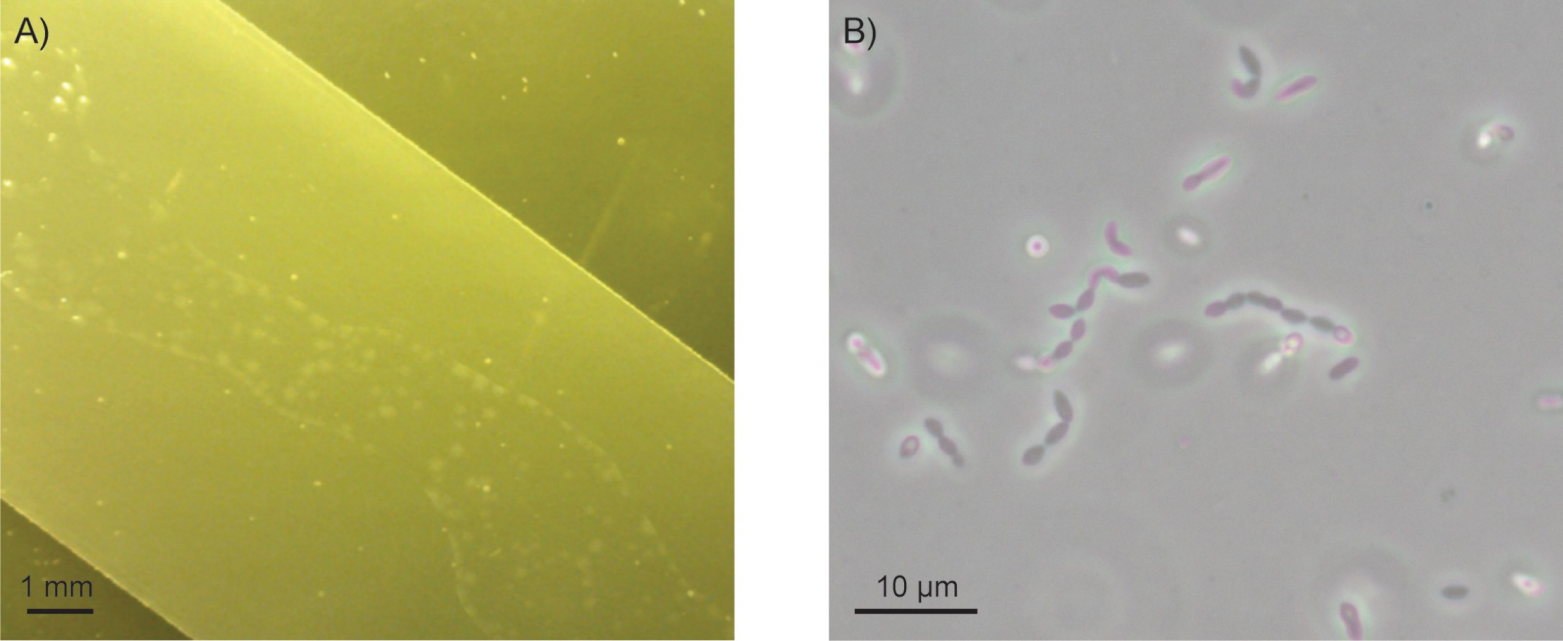
Photographic images. A) A close-up photograph of microbial growth on a PAO chip that was placed on top of CP agar. The chip was inoculated with 10-fold diluted cryo-preserved faecal sample from patient 188. The area that was inoculated is visualized as a smear in which individual microbial colonies can be distinguished. The white dots in the picture represent air bubbles in the agar medium. B) A light microscopy picture of the strain corresponding to OTU3088 that was isolated by a targeted approach. The strain shares 99% 16S rRNA gene identity with *S*. *intestinalis* BR72^T^.

Undiluted, 10-fold diluted and 100-fold diluted faecal suspension from 20 patients under the SDD regime was inoculated in duplicate on PAO chips that were placed on top of GAM and CP agar media that either did or did not contain the SDD antibiotic cocktail. Agar media inoculated with the lowest dilution of faecal material and in absence of antibiotics always yielded confluent growth on GAM media, whereas on CP media confluent growth was observed on 34 of 40 PAO chips (S4 Table). Growth on most PAO chips (371 of 480) was confluent as opposed to colonies that could be visually distinguished (Fig 1A). In general, a smaller number of colonies developed on media if the faecal material was inoculated at a higher dilution, if the media contained the SDD cocktail of antibiotics, and if the faecal material was inoculated on CP media. Faecal inocula (technical duplicates) and a selection of PAO chips with bacterial growth, including chips inoculated with undiluted and 100-fold diluted faecal suspensions, as well as chips inoculated with 10-fold diluted suspensions from patients 131, 148 and 210, were analysed by 16S rRNA gene amplicon sequencing. This amounted to a total of 324 samples. The NG-tax pipeline was used to process the sequence data of our samples as well as mock communities with known composition that were added to each library [38]. The average Pearson correlation of theoretical and observed OTU-level composition of the included mock communities was 0.82 (min-max 0.77–0.88), supporting the reliability of the applied approach (S5 Table). Samples with 0 reads (n = 4) assigned were removed from all further analysis yielding 319 samples with an average read depth of 40,999 ± 49,592 (s.d.) reads (S6 Table), and 3,832 assigned OTUs.

### Comparison of bacterial growth communities

#### Bacterial diversity

**Fig 2:**
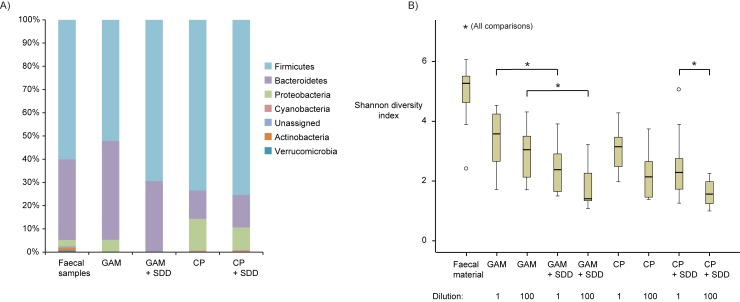
Diversity of the bacterial communities. A) Bacterial phyla that were detected in the faecal samples of 20 intensive care patients and in their corresponding growth communities on GAM and CP agar media. Growth on these media was further subdivided based on the addition of the SDD antibiotics. Phyla with a relative abundance <0.5% are not shown. The relative abundance values are based on the combined reads of all samples in the different experimental groups. B) Boxplots depicting the distribution of Shannon diversity values of bacterial communities in the different experimental groups. Asterisks indicate that Shannon values of bacterial communities in experimental groups were significantly different (*p* = <0.05) based on the two-tailed t-test. Medium (i.e. GAM vs. CP) did not significantly affect Shannon values of bacterial growth communities.

Averaged across all faecal samples, the bacterial phyla detected at the highest relative abundance were *Firmicutes* (60.5% ± 23.5 [s.d.]), *Bacteroidetes* (33.9% ± 25.3), *Proteobacteria* (2.60% ± 6.40), *Actinobacteria* (1.56% ± 5.00) and *Verrucomicrobia* (0.49% ± 1.22) (Fig 2A). *Firmicutes* and *Bacteroidetes* were detected at the highest relative abundance in the corresponding growth communities on GAM and CP media, together comprising on average >80% of the bacterial growth communities. On GAM agar without antibiotics, *Proteobacteria* on average constituted 5.10% ± 16.7 of the communities whereas on GAM agar with the SDD cocktail (GAM-SDD), proteobacterial relative abundance was 0.03% ± 0.14. Similarly, on CP-SDD media, *Proteobacteria* showed lower relative abundance (a decrease from 13.6% ± 27.7 to 9.80% ± 2.92) than on CP media without antibiotics. Notably, *Cyanobacteria* were not detected in the faecal samples or on GAM media, but they were detected on CP media averaging 0.51% ± 6.40 relative abundance. The average Shannon diversity of faecal samples was significantly higher than that of growth communities grouped by medium, addition of antibiotics or dilution (two-tailed t-test, *p* = <0.01 for all comparisons) (Fig 2B). Lower Shannon diversity values were also observed in growth communities inoculated with more diluted faecal samples. However, this difference was only significant between communities on CP-SDD agar that was inoculated with undiluted and 100-fold diluted faecal sample (two-tailed t-test, *p* = 0.01). The addition of the SDD antibiotics significantly reduced the Shannon diversity on GAM media (*p* = <0.01) but not on CP media. Differences in OTU richness and whole-tree phylogenetic diversity between the sample groups followed the same trends as differences in Shannon diversity i.e. higher values were obtained for faecal samples and lower values were obtained if media were inoculated with more diluted faecal material or included antibiotics (S1 Fig). However, surprisingly, a higher dilution of the faecal inoculum did not affect whole-tree phylogenetic diversity on CP media without antibiotics (*p* = 0.81). Canonical correspondence analysis (CCA) of OTU-level data from all bacterial communities (faecal inoculates and cultivable fractions) indicated that cultivation medium and presence/absence of antibiotics could explain in total 3.69% of the variation in bacterial composition (explanatory variable faeces; 1.36%, GAM agar; 0.89%, GAM-SDD agar; 0.73%, CP agar; 0.71%, *p* = 0.002). As such, most variation in the compositional data reflects inter-individual differences. However, bacterial growth on CP agar was not found to be significantly affected by the addition of the SDD cocktail of antibiotics. The dilution factor of faecal inocula was also evaluated as explanatory variable but was found to not affect bacterial composition.

#### Inter-individual differences in communities

**Fig 3:**
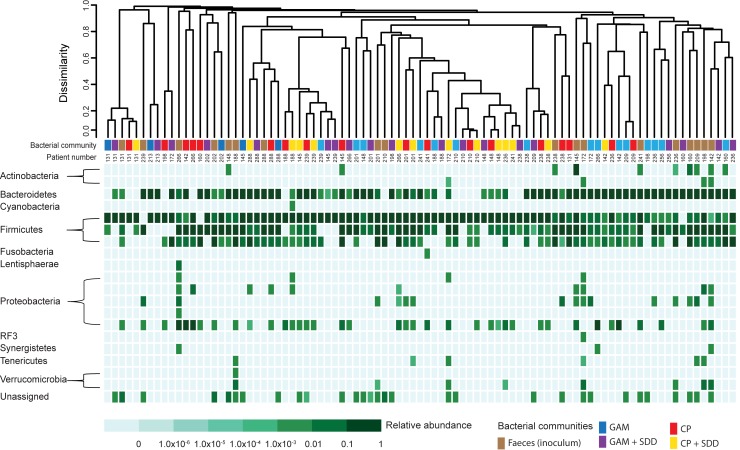
Hierarchical clustering using Bray-Curtis dissimilarity based on 16S rRNA gene amplicons generated from faecal samples of intensive care unit patients and corresponding biomass retrieved from PAO chips on GAM and CP agar. Growth on these media was further distinguished based on the addition of the SDD antibiotics. Hierarchical clustering was performed at the OTU-level. The heatmap corresponds to relative abundance values of class-level phylogenetic groups. For clarity, only communities derived from undiluted faecal samples, and only one of each biological (regarding growth communities) or technical (regarding faecal communities) replicate, was included in the dissimilarity tree.

While bacterial communities grouped by experimental variables (i.e. medium or addition of antibiotics) showed significant differences, we were also interested in potential differences between patients. Hierarchical clustering of OTU-level data using Bray-Curtis dissimilarity indicated high dissimilarity between individual faecal samples with mutual dissimilarity values being >0.8 in all but two cases (Fig 3). Growth communities derived from only 9 of 20 patients all clustered together indicating that other factors besides inoculum influenced bacterial growth. For example, growth communities of patient 131 all clustered together with mutual dissimilarity values <0.25 whereas for growth communities of patient 236 dissimilarity values exceeded 0.8. Moderate clustering by medium and presence/absence of antibiotics confirmed that cultivation conditions affected growth as was shown before by CCA. We used Bray-Curtis dissimilarity to evaluate to what extent bacterial growth communities differed from the faecal samples from which they were derived (S2 Fig). The Bray-Curtis dissimilarity indices ranged from 0.49 to 1.00, with large inter-individual differences. The dissimilarity between faecal samples and their respective growth communities was not significantly affected by the applied medium, antibiotics and/or dilution of the faecal inoculum.

#### Effects of media composition and antibiotics on bacterial growth

In the following, we aimed to identify OTUs that were enriched as a result of specific cultivation conditions. We fitted linear mixed-effect models on OTU-level data so that differences between individual patients could be taken into account (S7 Table). A total of 35 OTUs were significantly enriched under the different cultivation conditions (Table 1). Considering Bonferroni-corrected p-values, a total of seven OTUs belonging to the families *Bacteroidaceae* (5 OTUs), *Staphylococcaceae* (1 OTU) and *Enterococcaceae* (1 OTU) were present in significantly higher abundance on GAM media as compared to the respective faecal samples. In contrast, on CP media, OTUs belonging to the families *Halomonadaceae* (2 OTUs), *Lachnospiraceae* (6 OTUs), *Ruminococcaceae* (1 OTU), *Streptococcaceae* (1 OTU), *Enterococcaceae* (3 OTUs), *Porphyromonadaceae* (2 OTUs), and *Oxalobacteraceae* (1 OTU) were enriched. A significantly lower abundance of *Ruminococcaceae* spp. (1 OTU), *Enterobacteriaceae* spp. (6 OTUs) and *Lachnospiraceae* spp. (4 OTUs) was detected on media supplemented with the SDD antibiotics in comparison to media without antibiotics, indicating that the antibiotics inhibited the growth of these bacteria.

**Table 1:** Linear mixed-effect models of OTU-level composition data of bacterial growth were applied to investigate which OTUs varied in abundance as a results of cultivation conditions.

		Taxonomy	
Enriched on	No. of OTUs	Family	Genus
GAM agar	5	*Bacteroidaceae*	*Bacteroides*
	1	*Staphylococcaceae*	*Staphylococcus*
	1	*Enterococcaceae*	*Enterococcus*
CP agar	2	*Halomonadaceae*	*Halomonas*
	6	*Lachnospiraceae*	Unspecified
	1	*Ruminococcaceae*	Unspecified
	1	*Streptococcaceae*	*Streptococcus*
	3	*Enterococcaceae*	*Enterococcus*
	2	*Porphyromonadaceae*	*Parabacteroides*
	1	*Oxalobacteraceae*	*Undibacterium*
Media (CP and GAM) without antibiotics	1	*Ruminococcaceae*	Unspecified
	6	*Enterobacteriaceae*	*Escherichia-Shigella*
	4	*Lachnospiraceae*	Unspecified
CP agar without SDD cocktail of antibiotics	1	*Ruminococcaceae*	Unspecified

Listed are the taxonomic affiliations of OTUs that were found to be enriched at Bonferroni corrected p-values of <0.05. Bacterial communities were cultivated on GAM agar and CP agar, both in the presence and absence of the SDD antibiotics.

### Growth of novel species

We further aimed to investigate whether novel species or members of taxa on the most wanted list were present in the cultivable fraction of the faecal samples. Thirteen high priority most wanted OTUs were detected in the growth communities; however, none of these OTUs was present at a relative abundance of >0.8% (S8 Table). Furthermore, three medium priority most wanted OTUs (OTUs 236, 172 and 288) were detected in the cultivable fraction with 1–3% relative abundance. Nevertheless, members of medium priority OTUs were not considered candidates for isolation because they shared 100% identity with strains that were previously isolated from non-human sources. Comparison of OTUs with the SILVA database of type strains yielded 16 OTUs with >2% relative abundance of which the OTU representative read shared <95% identity with the 16S rRNA gene sequence of the closest type strain (Table 2). Therefore, these OTUs were considered to i) potentially represent novel species, and ii) to be sufficiently abundant for isolation by colony picking. Among these 16 OTUs, OTUs 3088, 322, 3797, 2642 and 2024 were considered prime candidates for targeted isolation based on their relative abundance on the PAO chips (>5%) and novelty. OTU3088 shared 93.4% identity with the closest type strain, that is, *Ruminococcus torques*, and was present on GAM-SDD agar at 49.8% relative abundance (S8 Table). OTU3088 was also detected on GAM, CP and CP-SDD agar media, albeit at a lower relative abundance. We detected three additional OTUs (OTUs 3067, 3103 and 3070) at >2% relative abundance of which the closest type strain was also *Ruminococcus torques*. Since these three OTUs were always detected in samples that contained OTU3088, and since their representative reads shared high nucleotide identity with OTU3088 (>99%), we considered that they may be derived from the same bacterial strains. The best hits in the SILVA type strain database of OTU322 and OTU3797 were *Hydrogenoanaerobacterium saccharovorans* and the chloroplast of the diatom *Thalassiosira pseudonana*, respectively, and both were detected at >10% relative abundance (OTU322, max. 23.9%; OTU3797, max. 13.2%). Both OTU322 and OTU3797 were only detected at >1% relative abundance on CP agar media in the absence of the SDD antibiotics. The representative read of OTU2642 shared 94.7% nucleotide identity with the 16S rRNA gene of *Bacteroides ovatus*. OTU2642 was only detected on GAM media at a maximum of 7.4% relative abundance. Finally, the closest type strain of OTU2024 was *Oscillibacter ruminantium*, and it was detected at >1% relative abundance exclusively on CP-SDD media.

**Table 2:** Here are shown the OTUs in the growth communities of which the representative read shares <95% identity with the closest 16S rRNA gene sequence in the SILVA database of type strains.

OTU ID	No. of samples	GAM/CP medium	SDD/NAB	Highest rel.ab. (%)	Detected in inoculum?	Closest type strain	Accession number	% identity
3088	30	GAM/CP	SDD/NAB	49.8	yes/no	*Ruminococcus torques*	L76604	93.4
322	15	CP	NAB	23.9	yes/no	*H*. *saccharovorans*	EU158190	89.3
3797	2	CP	NAB	13.2	no	*T*. *pseudonana* (chloroplast)	EF067921	85.9
2642	4	GAM	NAB	7.4	yes	*Bacteroides ovatus*	EU136682	94.7
2024	2	CP	SDD	5.6	yes	*Oscillibacter ruminantium*	JF750939	91.1
2026	3	CP	SDD	5.5	yes	*Oscillibacter ruminantium*	JF750939	91.1
2082	1	GAM	SDD	3.9	no	*Coprobacter fastidiosus*	JN703378	94.7
2724	1	GAM	SDD	3.8	no	*Bacteroides faecis*	GQ496624	96.7
2985	2	CP	NAB	3.6	no	*Clostridium clostridioforme*	M59089	96
3067	4	GAM	NAB	3,0	yes	*Ruminococcus torques*	L76604	93
3103	4	GAM	NAB	2.8	yes	*Ruminococcus torques*	L76604	93
2252	3	GAM	SDD/NAB	2.6	no	*Bacteroides nordii*	EU136693	95
3375	2	GAM	NAB	2.2	yes	*Coprococcus comes*	EF031542	97
2884	2	GAM	NAB	2.2	no	*Clostridium bolteae*	AJ508452	96
3070	5	GAM	NAB	2.2	yes/no	*Ruminococcus torques*	L76604	93
2893	2	GAM	NAB	2.0	no	*Clostridium bolteae*	AJ508452	96

The relative abundance of the listed taxa was ≥2% on at least one PAO chip.

### Targeted cultivation and antibiotic susceptibility profiling

To provide proof of concept, we aimed to isolate strains corresponding to OTUs 3088 and 2024 as they were considered prime candidates for isolation based on novelty. Furthermore, the fact that these OTUs grew on media containing the SDD antibiotics suggested they may be antibiotic resistance reservoir species. We prepared dilution series of the growth fractions in which these OTUs were most enriched and subsequently inoculated the diluted samples under the exact conditions that previously yielded enrichment of the target OTUs. This experiment, however, did not yield isolation of the target OTUs. Therefore, the protocol was repeated using the original faecal samples as inocula instead of the enriched growth fractions. By this method we isolated a member of OTU3088, which was demonstrated by the fact that the representative read of OTU3088 shared 100% identity with the 16S rRNA gene Sanger read of our isolate (Fig 1B). BLASTn of the 16S rRNA gene read of the isolate against the NCBI ribosomal 16S RNA sequences database showed that closely related strains (98–99% nucleotide identity over 1,399 nucleotides) have recently been isolated in four other laboratories. One closely related strain that was isolated from human faeces was recently published as a novel species named *Sellimonas intestinalis* BR72^T^ [49]. As such, our isolate was named *S*. *intestinalis* HF3088. Antibiotic susceptibility profiling using the ATB ANA EU (08) panel showed that *S*. *intestinalis* HF3088 was resistant only to metrodinazole (4 μg/ml) and imipenem (8 μg/ml) according to the CLSI guideline. The strain was not resistant to any of the 14 other antibiotics in the panel that also included cefotaxime (10 μg/ml) (S9 Table). In addition, microdilution testing yielded MIC values of 4 μg/ml for metronidazole and 64 μg/ml for imipenem. The MIC values for the other antibiotics tested by microdilution were 16 μg/ml for tetracycline, 0.5 μg/ml for ampicillin, and 16 μg/ml ceftriaxone (S9 Table), indicating that the strain was susceptible to these antibiotics.

## Discussion

In this study, we investigated the cultivability of anaerobic human faecal bacteria in order to isolate strains that can serve as reservoirs for antibiotic resistance. Therefore, faecal samples from 20 intensive care patients who received SDD therapy were inoculated anoxically on PAO chips placed on top of poor and rich agar media, including media supplemented with the SDD antibiotics. Faecal inocula and associated bacterial growth communities were analysed by 16S rRNA gene amplicon sequencing.

Selection of potential new reservoir species was first attempted by comparing the cultivated species with the strains on the most wanted list that comprises human-associated bacteria of which the genome has not yet been sequenced and that are grouped into priority classes based on novelty [24]. We did not detect high priority taxa at >1% relative abundance in the growth communities. Medium priority taxa were detected at 1–3% relative abundance but they shared 100% 16S rRNA gene sequence identity with strains for which the genome has been already sequenced. Therefore, due to the low relative abundance and/or high similarity to previously genome-sequenced bacteria, the detected medium and high priority taxa were not further considered prime candidates for isolation. In our second attempt, we compared the OTU centroid reads of cultivated bacteria with cultured strains in the SILVA type strain database. This yielded 16 OTUs that shared <95% nucleotide identity with type species, all of which exceeded 2% relative abundance. Based on their novelty, members of these OTUs were considered candidates for isolation. Although their prevalence in the gut microbiota is expected to be <20%, i.e. they are not on the most wanted list, understanding the biology of such populations might be highly relevant in a more personalized approach, where individual-specific microbiota signatures are considered key to success [50].

Fifteen out of these 16 novel OTUs detected in the growth communities represented species belonging to the *Firmicutes* (11 OTUs) and *Bacteroidetes* (4 OTUs). Notably, the remaining novel OTU (OTU3797) was detected on CP agar at a maximum relative abundance of 13.2%, and shared 85.9% nucleotide identity with the best hit in the SILVA type strain database, namely the chloroplast of *T*. *pseudonana*. Chloroplasts are thought to have originated from *Cyanobacteria* [51], and this finding might suggest the growth of a eukaryote capable of photosynthesis. This OTU was detected in five growth communities, exclusively on CP agar without antibiotics, and exclusively on PAO chips inoculated with faecal sample from two patients. While this strengthens the case for microbial growth, we cannot exclude (environmental) contamination. *T*. *pseudonana* has been shown to grow at a wide range of salinities and temperatures [52], and to have anaerobic capabilities [53].

OTU3088, which represented a novel member of the *Firmicutes*, was detected in samples of nine different patients. Furthermore, on one PAO chip OTU3088 was detected in the presence of the SDD antibiotics at a relative abundance of 49.8%. As such, it was selected for targeted isolation, which was achieved on GAM-SDD media (Fig 1B). After isolation, the 16S rRNA gene of the isolate was found to share 99% nucleotide identity with the recently published *S*. *intestinalis* BR72^T^ [49], and high identity (98–99%) with three other strains recently isolated from the human gut (accessions KT156811, LN828944 and AY960564). Therefore, even though a close relative of our isolate has been recently described, these results demonstrate that high-throughput cultivation-based screening can be used to isolate novel antibiotic resistant bacteria by a targeted approach.

Antibiotic susceptibility profiling of the isolated strain, which was named *S*. *intestinalis* HF3088, showed it to be resistant to imipenem and metrodinazole. As such, the strain is a candidate to be further analysed as resistance reservoir (e.g. by genome sequencing). *S*. *intestinalis* HF3088 in pure culture was not resistant to cefotaxime. This is unexpected considering that both during initial cultivation as well as during resistance testing of the pure isolate, the concentration of this antibiotic in the medium was 10 μg/ml. However, it should be noted that antibiotics may be broken down by adjacent bacteria on the PAO chip, and hence not all bacteria that grow are by definition resistant. For example, cefotaxime may be degraded through the secretion of a β-lactamase [54, 55]. We could hypothetise that the PAO chip acts as a buffer between the agar medium and the chip surface, thereby reducing the effective concentration of the antibiotic.

Besides OTU3088, five other novel OTUs were found to grow in the presence of the SDD antibiotic cocktail, and as such are additional candidates for isolation and characterization (Table 2). The lack of success in the isolation of a representative of novel OTU 2024 could have resulted from the dependence on microbe-microbe interactions [56]. Out of the 16 novel OTUs detected on agar media, only OTU (OTU3088) was detected on both GAM and CP media at >2% relative abundance. This indicates that the number of target species for isolation might be increased by including different media.

Our results showed that novel human-associated bacteria can still be cultivated using conventional methods. The extent to which novel bacteria can still be isolated by conventional methods was shown recently by Browne and co-authors in an experiment in which they isolated ~4,000 pure culture bacterial strains from faeces of six human individuals. The authors found that these isolates comprised as much as 96% of the bacterial abundance at the genus level and 90% of the bacterial abundance at the species level based on average relative abundance across faecal samples of the six individuals from whom the samples had been used in this study [57].

We also investigated bacterial growth not pertinent to the isolation of novel species. We showed that the composition of bacterial growth communities was significantly impacted by medium and by supplementation of media with antibiotics. The cocktail used in SDD therapy contains antibiotics that are predominantly active against Gram-negative bacteria and fungi [58], and was designed in order to eradicate potentially pathogenic bacteria from the gut without harming the anaerobic microbiota [25, 29]. Indeed, we found that six OTUs belonging to the genus *Escherichia*, one of the SDD target taxa, was present at significantly lower relative abundance on media containing the SDD cocktail. However, we also found that five OTUs belonging to the families *Ruminococcaceae* and *Lachnospiraceae* was present at significantly lower relative abundance in the presence of the SDD antibiotics. Members from these families are Gram-positive and lack aerobic respiration [59], and are therefore collaterally affected by the application of the SDD antibiotics that aim to lower the risk of infection with Gram-negative aerobic opportunistic pathogens [25]. We found that the similarity between the communities in the faecal inocula and the associated growth communities varied extensively between patients. We cannot exclude that this resulted from differences in viability between cryo-preserved faecal samples. Alternatively, it may have resulted from inter-individual differences in gut microbiota composition.

In conclusion, we have shown that high-throughput screening of growth communities for bacterial resistance can guide targeted isolation of potential reservoir species. The fact that a member of one novel antibiotic-resistant OTU (OTU3088) was successfully isolated demonstrates the viability of the approach. Follow-up isolation and characterization will be required to analyse the role of previously uncultivated species in the dissemination of resistance genes in the gut microbiota, including the transfer to potential pathogens.

## Supporting information

S1 Fig **Boxplots depicting the distribution of no. of OTUs (panel A) and phylogenetic diversity values (Panel B) of bacterial communities in different experimental groups.** The experimental groups are the faecal inocula and their corresponding growth communities on GAM and CP agar media. Growth on these media was further subdivided based on the addition of the SDD antibiotics. Asterisks indicate that these richness and diversity values of bacterial communities in experimental groups were significantly different (*p* = <0.05) based on the two-tailed t-test.
(EPS)

S2 Fig The bacterial communities in the faecal samples of 18 intensive care patients were compared to the corresponding growth communities on GAM agar and CP agar by calculating the Bray-Curtis dissimilarity indices of OTU-level taxa. Growth on these media was further distinguished based on the addition of the SDD antibiotics. Per patient the Bray-Curtis dissimilarity indices are sorted from higher to lower values.
(EPS)

S1 Table This table provides the following metadata regarding the patients of which faecas was sampled: The patient identification number (ID), his/her admission date, the date the sample was taken that was inoculated on the PAO chip, and the number of days after admission that this sample was taken. (XLSX)

S2 Table Here is shown which samples were included in each library and which primers were used. Panel A: the primers that were used for 16S rRNA gene amplicon sequencing. Panel B: read data were obtained from five different 16S rRNA gene amplicon MiSeq sequencing libraries. The metadata of the samples are given. SDD stands for the SDD cocktail of antibiotics i.e. tobramycin, polymyxin E, amphotericin B and cefotaxime. NAB stands for No AntiBiotics. The data were deposited under project accession PRJEB27463.
(XLSX)

S3 Table A mock community was included in every library that was sent for 16S rRNA gene amplicon sequencing. This table shows the OTU-level phyla that were present in the mock community.
(XLSX)

S4 Table This table gives information about the growth observed on each PAO chip at the time that the growth communities were harvested. Supplementary Table S3 Faecal samples of 20 patients were inoculated on PAO chips in duplo. Communities on GAM agar were harvested after 48 h and those on CP agar after 72 h. C stands for confluent growth.
(XLSX)

S5 Table Mock communities were included in libraries that was sent for 16S rRNA gene amplicon sequencing to examine the quality of the sequencing. Panel A: Pearson correlation coefficients between OTU-level taxa detected in the mock community samples and the theoretical mock community. Panel B: relative abundance of OTU-level taxa that were detected in the mock community samples. The final column shows the theoretical composition of the mock community.
(XLSX)

S6 Table This table gives information about the number of reads that were obtained for each sample by 16S rRNA gene amplicon sequencing (MiSEQ). Faecal samples of 20 patients were inoculated on PAO chips on top of agar media in duplo. Communities on GAM agar were harvested after 48 h and those on CP agar after 72 h.
(XLSX)

S7 Table Linear mixed-effect models of OTU-level composition data of bacterial growth were applied to investigate which OTUs varied in abundance as a results of cultivation conditions. This table lists the taxonomic affiliations of OTUs that were found to be enriched at FDR-corrected p-values of >0.05. Bacterial communities were cultivated on GAM agar and CP agar, both in the presence and absence of the SDD cocktail of antibiotics.
(XLSX)

S8 Table This table shows the OTUs that were detected in the different samples. Faecal samples of 20 patients were inoculated on PAO chips in duplo. The bacterial growth communities were analysed by 16S rRNA gene amplicon sequencing.
(XLSX)

S9 Table *S*. *intestinalis* HF3088, the strain that was obtained in pure culture, was tested for antibiotic resistance. Table A shows the results of the ANA EU(08) test, table B shows the result of the microdilution test, and table C shows the breakpoints for resistance that are recommended by CLSI and EUCAST.
(XLSX)

S1 Text Representative reads of the OTUs were compared by Blastn to the 16S rRNA genes in the SILVA database of cultured type strains. This text file contains the command of the BLAST search as well as the custom Perl script that parsed the BLAST output and tabulated the relative abundance of the OTUs and their distribution across samples.
(TXT)

S2 Text Representative reads of the OTUs were compared by Blastn to the the 16S V1-V3 sequence data of the most wanted OTUs. This text file contains the command of the BLAST search as well as the custom Perl script that parsed the BLAST output and tabulated the relative abundance of the OTUs and their distribution across samples.
(TXT)
